# Case report: Identification of a novel heterozygous germline *ERCC2* mutation in a patient with dermatofibrosarcoma protuberans

**DOI:** 10.3389/fonc.2022.966020

**Published:** 2022-08-10

**Authors:** Qing Zhang, Yongzhi Ju, Xia You, Tingting Sun, Yi Ding

**Affiliations:** ^1^ Department of Orthopaedic Oncology, Beijing Ji Shui Tan Hospital, Peking University, Beijing, China; ^2^ The Medical Department, Jiangsu Simcere Diagnostics Co., Ltd, Nanjing, China; ^3^ The Medical Department, Nanjing Simcere Medical Laboratory Science Co., Ltd, Nanjing, China; ^4^ The State Key Lab of Translational Medicine and Innovative Drug Development, Jiangsu Simcere Diagnostics Co., Ltd, Nanjing, China; ^5^ Department of Pathology, Beijing Ji Shui Tan Hospital, Peking University, Beijing, China

**Keywords:** DFSP (dermatofibrosarcoma protuberans), ERCC2 gene, sarcoma, NGS - next generation sequencing, xeroderma pigmentosa

## Abstract

Dermatofibrosarcoma protuberans (DFSP) is a kind of soft tissue sarcoma, mostly occurs in the trunk, followed by proximal extremities and head and neck. Surgical resection is the most important treatment for DFSP, but the local recurrence rate of DFSP is high. Except reported specific chromosomal tran7slocations occurred in DFSP, the association between DNA repair gene mutations and DFSP still unknown. In this report we found a 19-year-old boy with DFSP carries a novel heterozygous germline *ERCC2* mutation, which belongs to the nucleotide excision repair (NER) pathway and genetic defects in *ERCC2* may contribute to the cancer susceptibility xeroderma pigmentosum (XP), Cocaine syndrome (CS), and trichothiodystrophy (TTD). Different mutations of the *ERCC2* gene can lead to diverse diseases, but there are no targeted therapies. In summary, our results enlarged the mutation spectrum of the DFSP patients. It also provides new insights into genetic counseling and targeted therapeutic strategies for patients with DFSP.

## Introduction

There are more than 50 subtypes of soft tissue sarcomas, 30% of which are associated with specific genetic alterations, including translocations (1). Several studies have reported associations between cancer risk and DNA repair gene polymorphisms in the nucleotide excision repair (NER) pathway. NER involves more than 20 proteins, including xeroderma pigmentosum (XP) factors (group A to F) and cockayne syndrome (CS) factors (A and B), whose inactivation can cause xeroderma pigmentosum (XP) or Cockayne syndrome (CS) (2).

*ERCC2* gene is a member of the nucleotide excision repair system (NER). *ERCC2* gene encodes xeroderma pigmentosum group D (XPD) protein, one of the subunits of the TFIIH complex, which plays an important role in nucleotide excision repair function and basic transcription (3). *ERCC2* gene mutations can lead to XP, CS, and trichothiodystrophy (TTD). XP is a hereditary disease, harbored heterozygous mutations in R638W and R616P of *ERCC2* which decreased helicase activity (3). Patients with XP are extremely sensitive to sunlight and have an increased risk of skin cancer (4, 5). More epidemiological studies have reported that *ERCC2* SNPs can increase the risk of skin cancer, lung cancer, breast cancer, ovarian cancer, and bladder cancer (6–8). *ERCC2* somatic mutation has a high incidence in bladder cancer, accounting for about 10%, and is related to chemotherapy response (9). Meanwhile, more studies have reported the importance of DNA damage repair gene mutation in predicting tumorigenesis and tumor treatment response.

Dermatofibrosarcoma protuberans is (DFSP) a rare cutaneous soft tissue sarcoma, accounting for approximately 1.8% of all soft tissue sarcomas and 0.1% of all tumors (10, 11). DFSP progress slowly, mostly showing skin-colored plaque. It expands slowly for months or years, and finally becomes a nodule, which is easy to be ignored in the early stage (12). The most common form of DFSP is adhesion to the dermis, but it can move freely to the deeper cortex, may also adhere to bone or fascia, and the tumor may fester or have pain (13, 14). Although about 85-90% of DFSPs are low-grade lesions, about 10-15% of DFSPs contain high-grade differentiated fibrosarcomatous, which is more likely to recurrence and metastasis (15, 16). Surgical resection is the most important treatment for DFSP, but the local recurrence rate of DFSP is high, and it less metastasizes to the distal end.

In this case, we found a heterozygous germline mutation *ERCC2* c.105+1 G > C may be related to the occurrence of DFSP.

## Case presentation

**Figure 1:**
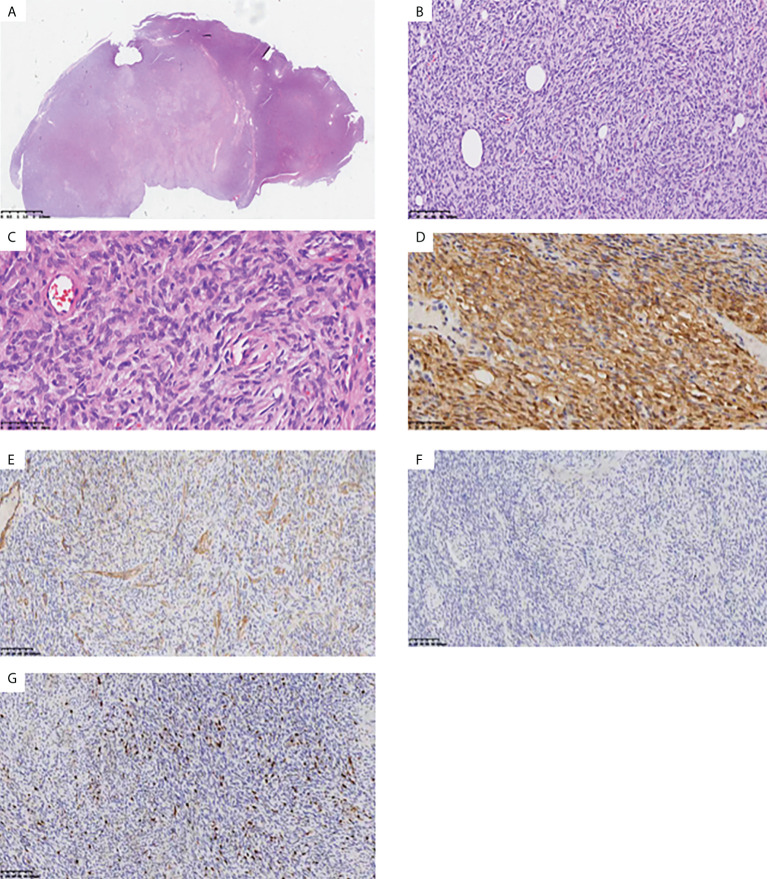
Histopathologic stains from the inner mass of the right back. HE staining results suggested spindle cell tumor. Low-power **(A)**, medium-power **(B)**, and high-power **(C)** view demonstrating a partially encapsulated nodular mass without dermal connection. Immunohistochemistry results suggested dermatofibrosarcoma protuberans: CD34 (+) **(D)**, SMA (-) **(E)**, S100 (-) **(F)**, Ki67+ 5-10% **(G)**.

**Figure 2:**
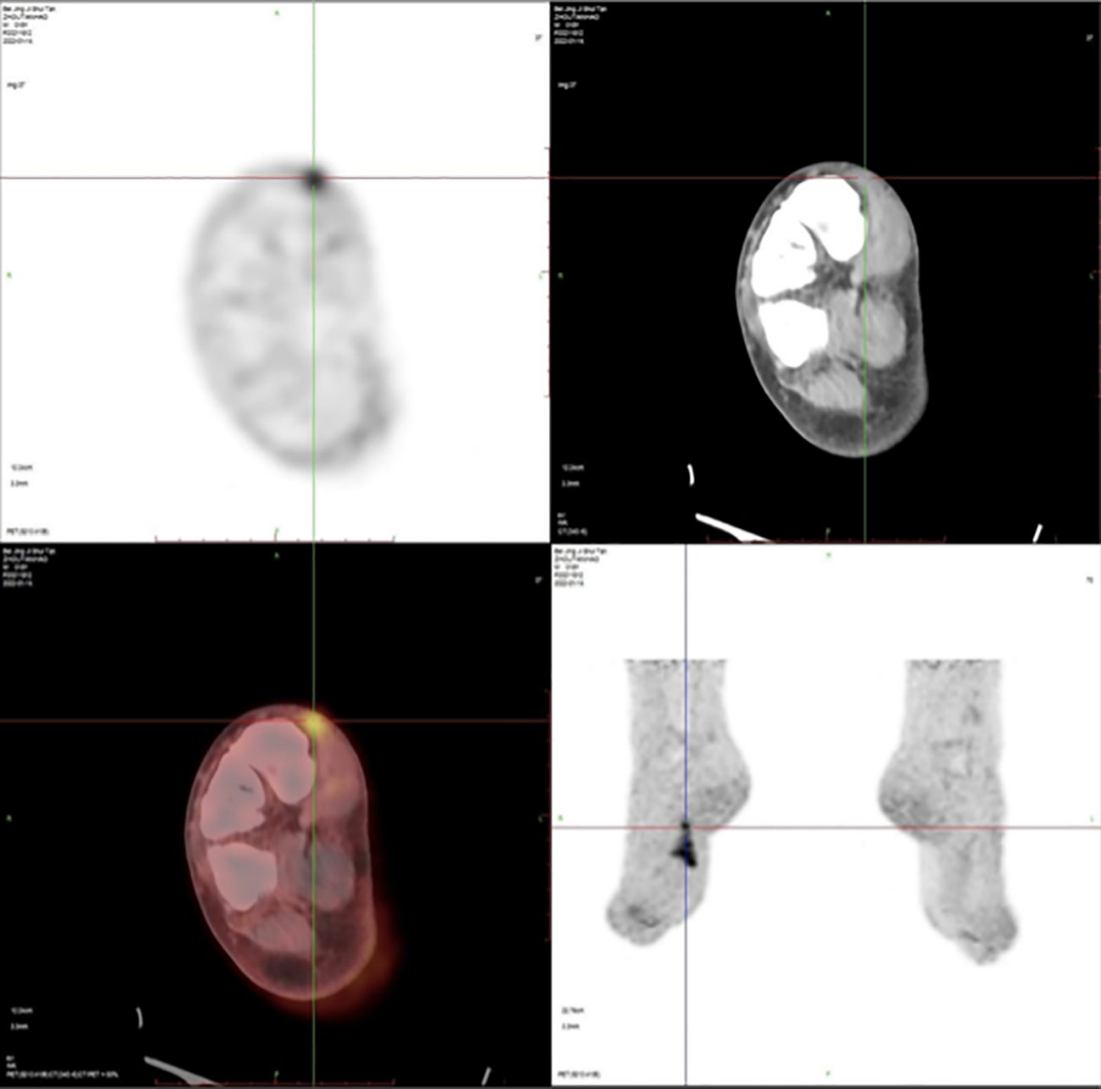
Positron emission tomography/computed tomography (PET/CT) imaging after unplanned resection of DFSP showed an increased radioactive uptake (SUVmax = 5.2) of soft tissue density as well as small nodules in the local subcutaneous soft tissue and have increased metabolic activity.

A 19-year-old boy had pain in his right toe during exercise in 2020. No bone destruction was found, and the pain was gradually relieved. The tubercle in the right toe proximal dorsal was found in June 2021 but without any treatments. In December 2021, due to the influence of enlarged nodules on wearing shoes, he went to the local hospital and underwent unplanned resection of the inner mass of the right back. The pathological examination was DFSP (**Figure 1**). The tumor is about 2×1cm, it was tough, tender, movable, had a clear boundary, and did not adhere to the deep tissue. PET/CT was performed in January 2022, and no distal organ or lymphatic metastasis was found (**Figure 2**). However, the patient’s local subcutaneous soft tissue thickening and small nodules have increased metabolic activity, which may be considered a possible postoperative residual or recurrence. At the same time, next gene sequencing (NGS) was performed on tissue and blood samples.

**Figure 3:**
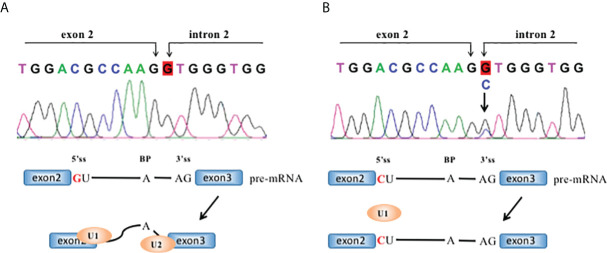
The *ERCC2* intron2 c.105+1 G>C mutation and its effect in RNA splicing. RNA splicing of wild-type **(A, B)** mutant *ERCC2*. Each intron has guanine and uracil (GU) at the 5′-end and adenine and guanine (AG) at the 3′-end. 5′ss: 5′ splice site; 3′ ss: 3′ splice site; BP, branch site.

The 551-gene NGS panel revealed a novel heterozygous germline *ERCC2* c.105+1 G > C mutation. This variation has never been reported in any database or any publications, such as the Exome Aggregation Consortium and 1000 Genomes Project. The variant is predicted to be pathogenic by MutationTaster and dbscSNV, which are used for functional prediction of splice variants. The splice-site variant in *ERCC2* (c.105+1 G > C) destroys a canonical splice donor site in intron 2, which may leading to an abnormal splicing of mRNA and affect its function (**Figure 3**). Loss-of-function variants in *ERCC2* are known to be pathogenic (3, 17–19). The ClinVar database records that the downstream *ERCC2*:c.594+2_594+5del is pathogenic/likely pathogenic. Based on the above analysis, we classified the variant as (likely) pathogenic according to the criteria of the American College of Medical Genetics and Genomics (ACMG).

**Figure 4:**
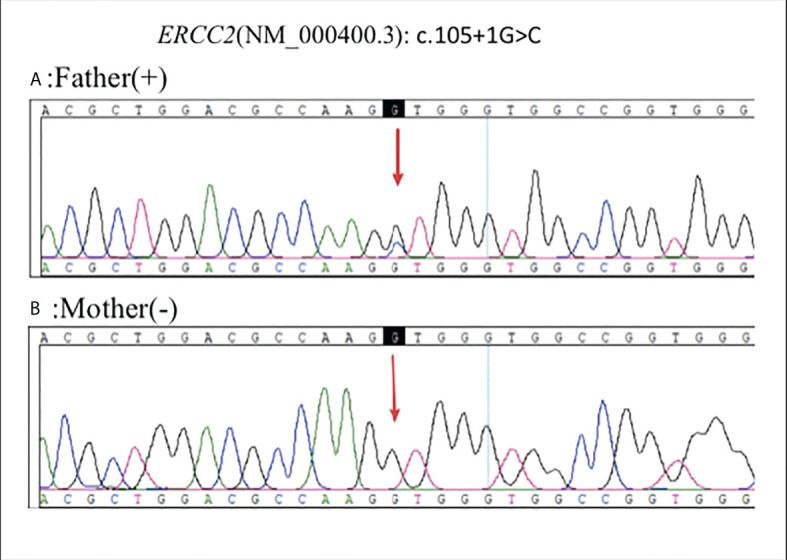
Sanger sequencing of blood samples from the patient’s parents was carried out for family verification: Father (+) **(A)**, Mother (-) **(B)**.

In April 2022, sanger sequencing of blood samples from the patient’s parents was carried out for family verification. The father’s diagnosis of polyliposarcoma also harbored this mutation, while variations were not detected in the unaffected mother (**Figure 4**).

## Discussion

Soft tissue sarcomas represent a heterogeneous group of rare tumors, 30% of which are associated with simple genetic alterations, including specific translocations. Studies have demonstrated that DNA repair plays a vital role in genomic maintenance to prevent carcinogenesis. *ERCC2* encodes the XPD protein, which is part of the TFIIH complex, has ATP-dependent DNA helicase activity, and belongs to the RAD3/XPD subfamily of helicases. *ERCC2* plays an important role in gene transcription and gene transcription-coupled nucleotide excision repair (20). Genetic defects in *ERCC2* may contribute to the cancer susceptibility XP, CS, and TTD.

XP patients are particularly sensitive to light, the risk of cutaneous tumors in sun-exposed areas of the body is 1000 fold that of normal individuals. The deficient NER system of XP patients cannot repair the UV damage, resulting in specific mutations accumulation in key regulatory genes in the skin, eye, ocular, and oral cells (21). Different mutations of the *ERCC2* gene can lead to diverse diseases, but there are no targeted therapies. With the increasing number of defects in genes regulating double-strand break (DSB) repair/homologous recombination (HR), such as *BRCA1/2* and mismatch repair (MMR) genes, other NER genes related to cancer pathogenesis, diagnosis, and treatment are also receiving increasing attention (22). There are *in vitro* experiments and case reports showing that the combination of different drugs with platinum-based chemotherapy (such as carboplatin and gemcitabine, irofulven, and cisplatin) can significantly improve the efficacy of patients with *ERCC2* mutations (22, 23). More studies are now exploring the association of platinum-based chemotherapy sensitivity with *ERCC2* mutations, which may be a potential therapeutic target.

In our case, the splice-site variant in *ERCC2* c.105+1 G > C identified in a young DFSP patient as well as his father who was diagnosis of polyliposarcoma. This variant was absent from all public DNA sequence databases. Previously reported mutations in the human *ERCC2* are mainly single residue changes and sometimes at adjacent which could case strikingly different genetic disorders, meanwhile, proved mutations in G47R (located in HD1 helicase motifI), T76A, D234N, G602D, and R683W showed greatly reduced helicase activity (24). Therefore, we can only study the significance of the mutation by itself. In principle, the RNA splicing process is tightly regulated, and may be dependent in part on whether the donor or acceptor site is affected. Aberrant RNA splicing is closely related to tumor development, growth, and progression to therapy-resistant tumors (25, 26). For example, a heterozygous splice-site mutation (c.619+1 G > C) at the 5’ end of intron 6 in *WDR77* is present in familial papillary thyroid cancers (27). The mutation caused exon 6 skipping, consequently leading to a frameshift and creating a premature stop codon for the new reading frame. It resulted in a shorter transcript and was only observed at very low levels, suggesting that the mutation triggers the nonsense-mediated messenger RNA (mRNA) decay (NMD) process. Splice-site mutation of *FLCN* in the 5’ end of intron 9 (c.1062+1 G > A) causing partial retention of intron, which was associated with Birt-Hogg-Dubé syndrome (28). A family with hereditary multiple osteochondroma (HMO) from Guangxi Province, China, harbored the pathogenic heterozygous c.1056+1 G > A mutation of *EXT1* (29). These studies suggest that guanine (G) mutation at the exon-intron junction was a very important event. In our case, the heterozygous c.105+1 G > C mutation destroys a canonical splice donor site in intron 2, which are most likely to cause changes in its function.

In summary, we identified a novel germline *ERCC2* mutation in a patient with DFSP. Our results enlarged the mutation spectrum of the DFSP patients. It also provides new insights into genetic counseling and targeted therapeutic strategies for patients with DFSP.

## Data availability statement

The original contributions presented in the study are included in the article/supplementary material. Further inquiries can be directed to the corresponding author.

## Ethics statement

Written informed consent was obtained from the individual(s) for the publication of any potentially identifiable images or data included in this article.

## Author contributions

YJ and XY prepared the manuscript and the literature search. TS reviewed and edited the manuscript. QZ treated and observed the patient. YD performed the histopathological and immunohistochemical examinations. All authors contributed to the article and approved the submitted version.

## Conflict of interest

Authors YJ, XY, and TS were employed by Jiangsu Simcere Diagnostics Co., Ltd and Nanjing Simcere Medical Laboratory Science Co., Ltd.

The remaining authors declare that the research was conducted in the absence of any commercial or financial relationships that could be construed as a potential conflict of interest.

## Publisher’s note

All claims expressed in this article are solely those of the authors and do not necessarily represent those of their affiliated organizations, or those of the publisher, the editors and the reviewers. Any product that may be evaluated in this article, or claim that may be made by its manufacturer, is not guaranteed or endorsed by the publisher.
